# Ontogeny, distribution and potential roles of 5-hydroxymethylcytosine in human liver function

**DOI:** 10.1186/gb-2013-14-8-r83

**Published:** 2013-08-19

**Authors:** Maxim Ivanov, Mart Kals, Marina Kacevska, Isabel Barragan, Kie Kasuga, Anders Rane, Andres Metspalu, Lili Milani, Magnus Ingelman-Sundberg

**Affiliations:** 1 Section of Pharmacogenetics, Department of Physiology and Pharmacology, Karolinska Institutet, Nanna Svartz väg 2, 17177 Stockholm, Sweden; 2 Estonian Genome Center, University of Tartu, Riia 23b, 51010 Tartu, Estonia; 3 Cancer Proteomics Mass Spectrometry, Department of Oncology-Pathology, Science for Life Laboratory, Karolinska Institutet Science Park, Tomtebodavägen 23A, 17165 Solna, Sweden; 4 Division of Clinical Pharmacology, Department of Laboratory Medicine, Karolinska University Hospital at Huddinge, Medicingatan 5, 14186 Stockholm, Sweden; 5 Estonian Biocentre, Riia 23b, 51010 Tartu, Estonia; 6 AQ2 Institute of Molecular and Cell Biology, University of Tartu, Riia 23b, 51010 Tartu, Estonia

## Abstract

**Background:**

Interindividual differences in liver functions such as protein synthesis, lipid and carbohydrate metabolism and drug metabolism are influenced by epigenetic factors. The role of the epigenetic machinery in such processes has, however, been barely investigated. 5-hydroxymethylcytosine (5hmC) is a recently re-discovered epigenetic DNA modification that plays an important role in the control of gene expression.

**Results:**

In this study, we investigate 5hmC occurrence and genomic distribution in 8 fetal and 7 adult human liver samples in relation to ontogeny and function. LC-MS analysis shows that in the adult liver samples 5hmC comprises up to 1% of the total cytosine content, whereas in all fetal livers it is below 0.125%. Immunohistostaining of liver sections with a polyclonal anti-5hmC antibody shows that 5hmC is detected in most of the hepatocytes. Genome-wide mapping of the distribution of 5hmC in human liver samples by next-generation sequencing shows significant differences between fetal and adult livers. In adult livers, 5hmC occupancy is overrepresented in genes involved in active catabolic and metabolic processes, whereas 5hmC elements which are found in genes exclusively in fetal livers and disappear in the adult state, are more specific to pathways for differentiation and development.

**Conclusions:**

Our findings suggest that 5-hydroxymethylcytosine plays an important role in the development and function of the human liver and might be an important determinant for development of liver diseases as well as of the interindividual differences in drug metabolism and toxicity.

## Background

There are substantial interindividual differences in many liver processes, including intermediary metabolism, protein synthesis, carbohydrate metabolism and the detoxification of drugs and other xenobiotics. With respect to variation in drug metabolism, transport and toxicity, much knowledge has been gained from studies investigating genetic factors responsible for interindividual differences. However, little is known regarding the role of epigenetic factors, such as DNA modifications and their influence on hepatic gene expression, function and interindividual variation [1].

The epigenetic DNA modifications include methylation and hydroxymethylation of cytosine. For many years, 5-methylcytosine (5mC) was believed to be the only epigenetic modification of genomic DNA, often referred to as 'the fifth base' of the genome. Recently, 5-hydroxymethylcytosine (5hmC) was discovered as a novel epigenetic factor in mammalian DNA that can have a stable effect on gene transcription and has hence been regarded as 'the sixth base' of the genome [2,3]. 5hmC originates from an enzymatic oxidation of 5mC by TET1, TET2 and TET3 proteins [2,4], which recognize their substrate 5mC by the amino-terminal CXXC zinc finger domain [5]. This reaction is dependent on Fe^2+ ^as well as α-ketoglutarate; the latter, in turn, is dependent on the activity of either isocitrate dehydrogenase IDH1 in the cytosol and peroxisomes or IDH2 and IDH3 in mitochondria [6,7]. Although other proteins may also have some significance in establishing the presence of genomic 5hmC, the TET and IDH proteins seem to play a very important role as determinants of the global 5hmC content.

Hydroxymethylcytosine can serve as an intermediate of active DNA demethylation if further oxidized by TET enzymes to 5-formylcytosine (5fC) and then to 5-carboxylcytosine (5caC), which in turn is removed by thymine-DNA glycosylase. The resulting abasic site is repaired by the base excision repair mechanism, producing unmethylated cytosine [8,9]. The level of 5fC was found to be extremely low in mouse embryonic stem cells and brain cortex (0.02 to 0.002% from total cytosine) [9,10]. Despite the recent progress in genome-wide mapping of 5fC in mouse embryonic stem cells, there is still no convincing evidence for its role as an epigenetic modification [11,12]. In contrast, 5hmC is 10 to 100 times more abundant than 5fC and can stably persist *in vivo*, thus serving as an epigenetic mark with unique regulatory functions.

Several functional differences have been demonstrated between the two DNA modifications 5mC and 5hmC. Firstly, the vast majority of CpG sites throughout the genome (except for those located in CpG islands and shores) are constantly methylated. In contrast, 5hmC can be found at only a relatively small subset of CpG sites. Furthermore, variable methylation correlating with gene expression has been described mostly in the promoter regions, whereas the genomic distribution of 5hmC is biased towards exonic regions. In addition, 5mC is, in most cases, a repressive epigenetic mark, whereas the presence of 5hmC correlates with active gene transcription [13-21]. The latter observation can be explained by the different affinity of 5mC and 5hmC for methylated DNA binding proteins. It was demonstrated that MeCP1, MBD1, MBD2 and MBD4 bind to methylated but not to hydroxymethylated loci [22,23], whereas MBD3 binds to 5hmC with even higher affinity than it does to 5mC [24]. Moreover, the composition of DNA binding and histone modifying proteins in 5hmC-containing loci can also be very different from that of methylated DNA, thus resulting in different gene expression outcomes [16,18-20,25].

Highly variable levels of 5hmC have been detected in many human and mammalian tissues and cell types with characteristic tissue-specific patterns [14]. 5hmC is especially enriched in brain tissue and in embryonic stem cells, where it constitutes up to 1 to 1.3% of the total cytosine content [14,17,26-29]. Furthermore, 5hmC has been shown to participate in neurodevelopment and has been associated with pluripotent cell states [30-32]. In cancers, such as prostate, breast, melanoma, glioma and colon carcinoma, the content of 5hmC is significantly decreased compared to normal tissue, is usually associated with decreased *TET *or *IDH *expression and, more importantly, can affect the tumor phenotype [7,14,33,34]. With respect to human liver, very little is known about 5hmC distribution and function.

In this study we demonstrate that adult human livers contain high levels of 5hmC, which are comparable to those found in the brain tissue. The analysis of genome-wide 5hmC hepatic distribution revealed that 5hmC is particularly enriched in coding regions of actively transcribed genes. In addition, the results show profound differences in both genomic distribution and global 5hmC content between fetal and adult liver samples. These differences were further pronounced by pathway analysis of genes enriched for 5hmC elements, which clearly showed consistency with liver development, thus suggesting that 5hmC may play an important role in hepatic development and function.

## Results

### Global 5mC and 5hmC content in fetal and adult human livers

**Figure 1:**
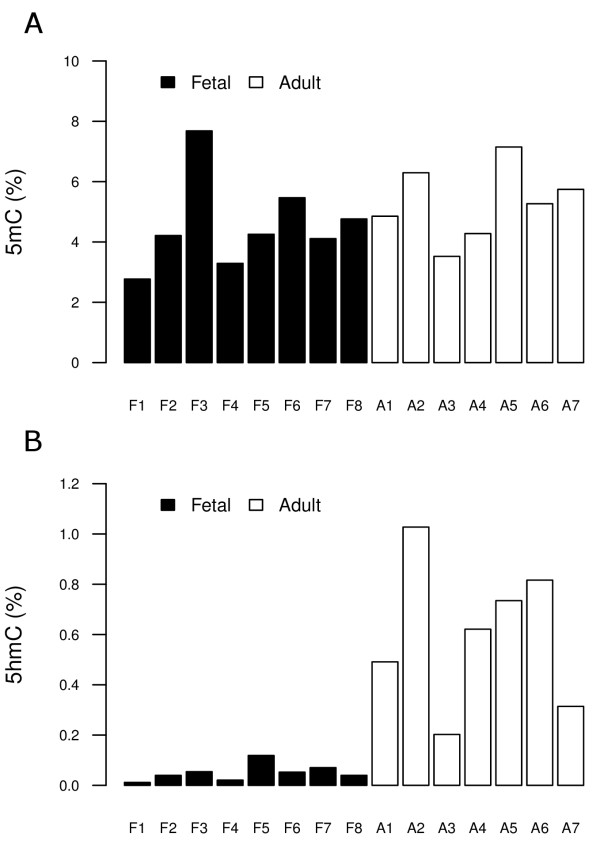
**Total 5mC and 5hmC in fetal and adult livers as determined by LC-MS**. The percentage of **(a) **5mC and **(b) **5hmC in relation to the total cytosine content in eight fetal and seven adult livers is shown.

Global levels of 5mC and 5hmC were determined in eight fetal and seven adult human livers using liquid chromatography-mass spectrometry (LC-MS) analysis, based on calibration curves with known 5mC and 5hmC content. The quantification range of 5hmC was from 0.0625 to 1.0% of total cytosine, suggesting our method is highly sensitive. The linear standard curve has excellent fit (R^2 ^> 0.999; Figure S1 in Additional file 1). Therefore, we used this calibration curve to determine global levels of 5mC and 5hmC in eight fetal and seven adult human livers. The 5mC and 5hmC contents of fetal and adult samples are shown in Figure 1 (for details, see Table S2 in Additional file 1). The median 5mC content was 4.2% of total cytosine in fetal livers and 5.3% in adult livers. By contrast, much less 5hmC was found in fetal livers compared to adult livers (Figure 1). All eight fetal samples had a 5hmC content lower than 0.125%, whereas the signal from six out of seven adult samples was above 0.2%, and the median 5hmC content for adults was 0.62% of total cytosine.

### Immunohistostaining of human liver samples

**Figure 2:**
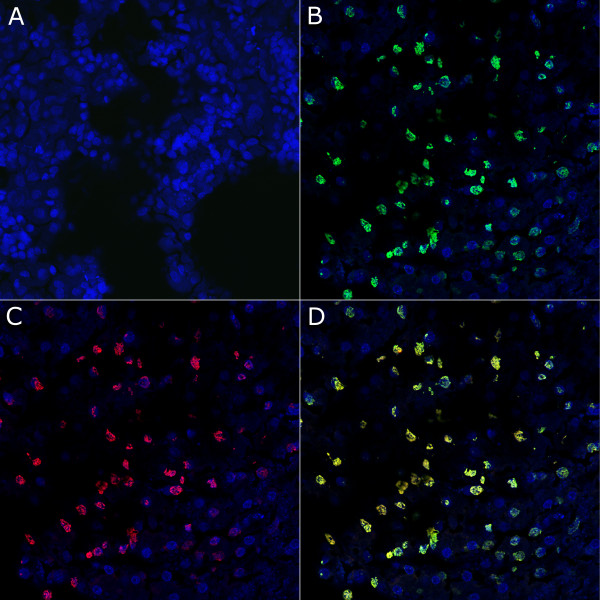
**Immunostaining of human adult liver for 5mC and 5hmC**. Representative stainings of 40 µM adult liver sections are shown. **(a) **Negative control without the primary antibody. **(b) **Staining with the monoclonal antibody to 5mC (green) and DAPI (blue). **(c) **Staining with a polyclonal antibody against 5hmC (red) and DAPI (blue). **(d) **Merge of 5mC and 5hmC staining and DAPI. As evident from (d), 5mC and 5hmC show similar patterns of intranuclear distribution, indicating that 5hmC, when present, tends to co-localize with 5mC in the hepatocyte nucleus.

In order to explore the distribution of 5hmC in the liver, we immunostained human liver sections for both this cytosine modification as well as for 5mC. As depicted in Figure 2, both 5mC and 5hmC were detected in the nuclei of most hepatocytes within the liver tissue sections. No significant difference regarding intranuclear localization between 5mC and 5hmC was observed. Three-dimensional analysis of the sections further revealed the abundant distribution of 5hmC to the majority of hepatocytes (data not shown).

### Quantification of *TET1-3 *and *IDH1-2 *transcripts

**Figure 3:**
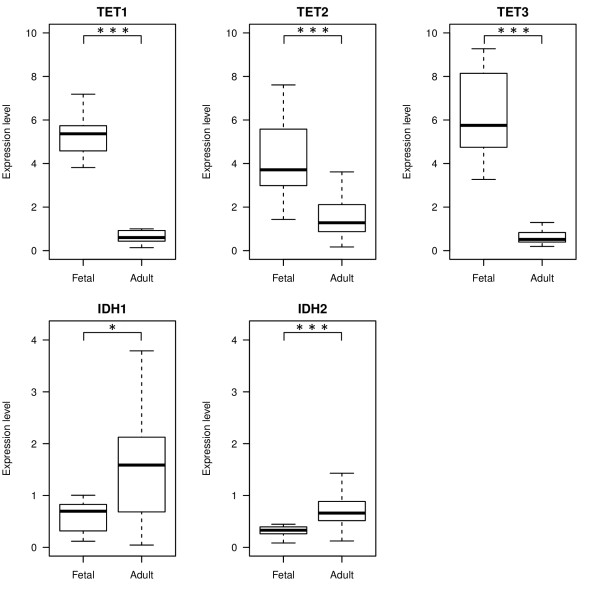
**Expression levels of the *TET1-3 *and *IDH1-2 *genes in fetal and adult livers**. Gene expression at the mRNA level was determined using quantitative RT-PCR and specific primers in samples from 14 fetal and 33 adult livers and normalized against *EIF2B2*. The boxplots show the relative gene expression in a log2-scale. The maximum length of each whisker is 1.5 times the interquartile range (IQR). The significance of differences is indicated by asterisks: *P < 0.01, ***P < 0.0001.

The observed differences in global 5hmC content between fetal and adult cohorts may be explained by differences in expression of the TET and/or IDH enzymes, which are involved in the conversion of 5mC to 5hmC. The mRNA levels of *TET1-3 *and *IDH1-2 *were assessed by quantitative RT-PCR in 14 fetal and 33 adult livers, including the samples investigated by LC-MS. Interestingly, the results showed a significant decrease in all *TET *genes (*P *< 0.0001) in adult livers when compared to fetal (Figure 3). However, the slight increase in *IDH1 *(*P *= 0.0024) and *IDH2 *(*P *< 0.0001) in adult samples may potentially explain the higher total 5hmC content in these livers compared to fetal 5hmC levels.

### Genome-wide profiling of 5hmC

To investigate the genome-wide distribution of 5hmC, we enriched the 5hmC-containing fraction of the DNA samples from the eight fetal and seven adult human livers. Both 5hmC-enriched DNA samples and their non-enriched counterparts were ligated with adapters, amplified and then subjected to next-generation sequencing (NGS), as described in Materials and methods. The main quality metrics of the NGS experiment as well as the number of called 5hmC peaks for each liver sample are listed in Table S3 in Additional file 1.

Overall, the fetal liver samples showed significantly fewer 5hmC peaks (11,366 to 32,522) covering a smaller portion of the genome (10 to 40 Mb) compared to adult samples (68,779 to 134,956 peaks covering 80 to 203 Mb). These results are in agreement with the finding by LC-MS that global 5hmC content is lower in fetal livers than in adults. Furthermore, the 5hmC peaks appear to be unevenly distributed among chromosomes, being over-represented on chromosomes 16, 17, 19 and 22 and under-represented on the sex chromosomes (Figure S2 in Additional file 1).

**Figure 4:**
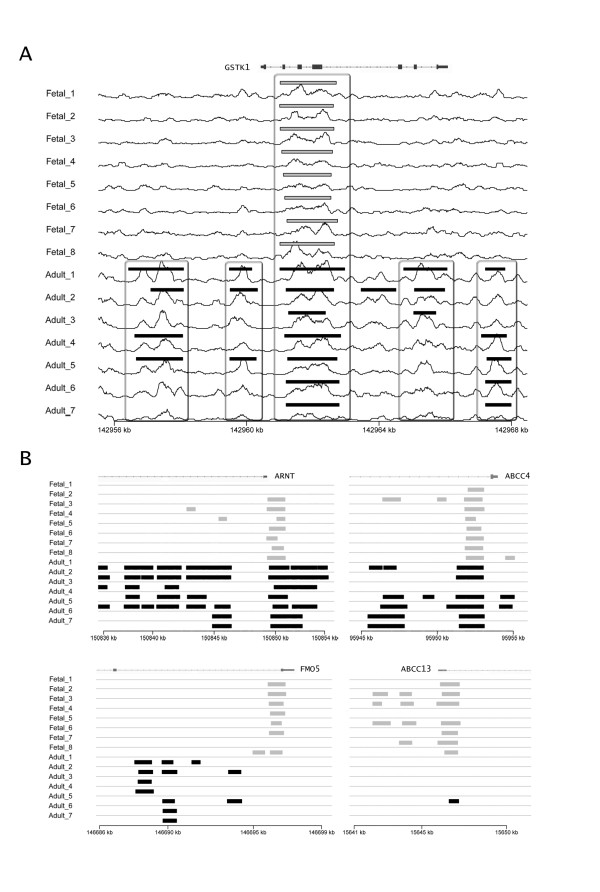
**The distribution of 5hmC peaks in five liver genes**. **(a) **A detailed view of the *GSTK1 *gene. For each 5hmC-enriched sample (y-axis), the read depth is plotted against the genomic coordinates in the given interval (x-axis). The 5hmC peaks called by the MACS software are shown by the short horizontal lines. The 5hmC blocks identified in this study are denoted by vertical rectangles. **(b) **Representative illustration of the genomic positions of 5hmC peaks identified in the genes *ARNT*, *ABCC4*, *FMO5 *and *ABCC13*. The fetal peaks are shown in gray and the adult peaks are shown in black.

When investigating the proportion of peaks that were shared by samples belonging to each cohort, a small fraction of peaks was shared by all fetal samples (2.5%) and all adult samples (8.3%; Figure S3 in Additional file 1). A large majority of the peaks were identified as unique peaks (54.4% in fetal and 36.1% in adult livers), suggesting high interindividual variability in the genomic distribution of 5hmC. On the other hand, a significant proportion of hydroxymethylation reproducibly appears at the same genomic locations, as seen when comparing 5hmC peaks shared by more than one sample. Examples of the distribution of 5hmC peaks in five different genes are shown in Figure 4. We focused on the continuous genomic intervals, or '5hmC blocks', where 5hmC occupancy was detected in at least two samples in a given cohort (Figure 4a). In other words, '5hmC blocks' represent genomic intervals that are prone to be hydroxymethylated in a given cohort (although not necessarily hydroxymethylated in every sample in this cohort). In contrast, the probability to detect 5hmC outside of 5hmC blocks is relatively low. Of the five genes illustrated in Figure 4, most had many more 5hmC blocks in adult livers, whereas in the *ABCC13 *gene, 5hmC blocks were mainly identified in fetal livers. The relation between this variability and the differences in gene expression between the livers has to await analyses in larger cohorts. The full lists of fetal (*n *= 29,917) and adult 5hmC blocks (*n *= 116,911) identified in this study are available in Additional files 2 and 3, respectively.

### Locus-specific validation of next-generation sequencing data

To validate our NGS data, we profiled 5mC and 5hmC content at single-base resolution for two randomly selected genomic intervals in four fetal and four adult liver DNA samples. The first interval (containing four CpG sites overlapping with a fetal-only 5hmC block) was located in intron 15 of the *DROSHA *gene, whereas the second one (containing seven CpG sites) was located in intron 2 of the *CDH2 *gene and manifested adult-only hydroxymethylation. The results of the single-base analysis are shown in Figure S5 in Additional file 1. The fetal hydroxymethylation in *DROSHA *(averaged over four CpG sites in each sample) could reach up to 35%, whereas in adult samples it did not exceed 4%. In contrast, fetal hydroxymethylation in *CDH2 *(averaged over seven CpG sites) was never higher than 5%, whereas in adult samples it varied between 49 and 56%. Thus, despite the single false positive and false negative 5hmC peaks identified for both genes, the 5hmC values obtained from the validation experiment are in good agreement with the NGS data.

### Genomic and functional localization of 5hmC

**Figure 5:**
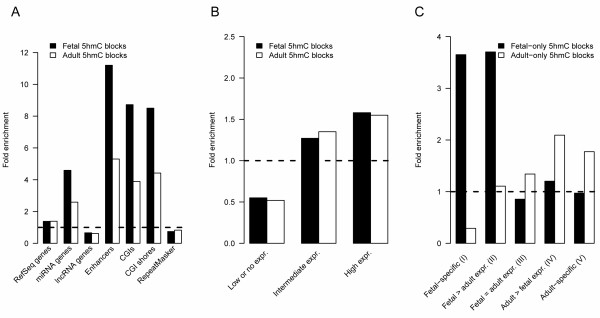
**Relative enrichment of 5hmC blocks in various genomic regions**. **(a) **The relative enrichment of fetal and adult 5hmC blocks in genomic features compared to H_0 _of random genomic distribution of 5hmC blocks. **(b) **The relative enrichment of fetal and adult 5hmC blocks in groups of genes manifesting low, intermediate or high gene expression. **(c) **Enrichment of fetal-only and adult-only 5hmC blocks in groups of genes manifesting different patterns of expression changes in liver development. The dashed line denotes the reference enrichment rate.

Next, we investigated the possible enrichment of 5hmC blocks in RefSeq genes, microRNA (miRNA) and long non-coding RNA (lncRNA) genes, hepatic enhancers, CpG islands (CGIs), CGI shores and repetitive sequences. We found that both fetal and adult 5hmC blocks are slightly enriched in RefSeq genes and strongly enriched in enhancers, miRNA genes, CGIs and CGI shores, but under-represented in lncRNA genes and repetitive regions (Figure 5a). Furthermore, the relative enrichment of 5hmC blocks in enhancers, miRNA genes, CGIs and CGI shores is approximately double in fetal samples compared to adults, whereas in RefSeq genes and lncRNA genes as well as in repetitive regions the rate of 5hmC enrichment remains nearly constant during liver development.

To investigate if cytosine hydroxymethylation in human liver correlates with active gene transcription, we analyzed how 5hmC blocks are distributed among genes with different expression levels. To this end, we quantified the genome-wide mRNA expression levels of 14 fetal and 86 adult liver samples (including all samples employed in the NGS study). The analyzed 17,771 RefSeq genes were divided into low, intermediate and highly expressed genes, separately for fetal and adult cohorts. The relative enrichment of 5hmC blocks in each of these three categories of genes is shown in Figure 5b. Both fetal and adult 5hmC blocks are under-represented in the genes with low expression or silent genes, and over-represented in genes with intermediate or high expression, suggesting a likely role for hydroxymethylation in active gene transcription.

### 5hmC localization during development

The observed profound differences in both global 5hmC content and the number of called 5hmC peaks between the fetal and adult liver samples suggest that 5hmC distribution is significantly modified during liver development throughout the genome. When comparing the fetal and adult hydroxymethylomes, we found 5,038 5hmC blocks present in fetal livers only and 96,097 blocks detectable only in adult samples. These fetal- and adult-specific 5hmC blocks were then investigated for their distribution among the following five gene groups: I) fetal-specific genes (expressed in fetal samples, but silent in adults; *n *= 464); II) genes expressed in fetal samples at least two-fold higher than in adults (*n *= 805); III) non-developmentally expressed genes (*n *= 8,731); IV) genes expressed in adult samples at least two-fold higher than in fetal samples (*n *= 1,079); and V) adult-specific genes (expressed in adult cohort, but silent in fetal cohort; *n *= 306). Relative enrichment values of genic fetal-specific and adult-specific 5hmC blocks in these groups of genes are shown in Figure 5c. As evident from these data, fetal-specific 5hmC blocks were seen strongly enriched in fetal expressed genes (groups I and II) but not in non-developmental (group III) or adult-specific genes (groups IV and V). Accordingly, adult-only 5hmC blocks are strongly under-represented in fetal-only genes (group I), but skewed towards genes that are exclusively expressed in the adult livers (groups IV and V). Hence, developmental patterns of hydroxymethylation correlate with developmental patterns of gene transcription in human livers.

### 5-Hydroxymethylation in relation to gene function

Finally, genes intersecting with fetal-only or adult-only 5hmC blocks were annotated to specific biological pathways using the GREAT software. Those genes that acquired hydroxymethylation in the adult state were found to be involved in active catabolic and metabolic processes, such as mRNA catabolic process, carbohydrate transport, fatty acid oxidation and lipid oxidation, which is well in line with the function of the adult liver. Meanwhile, the genes containing fetal-only 5hmC blocks were more specific to pathways for differentiation and development, such as regulation of cell differentiation, stem cell development, and establishment or maintenance of cell polarity. The top 10 biological processes for these two groups of genes are given in Table 1.

**Table 1:** Functional annotation of developmentally hydroxymethylated regions

	Binomial test			Hypergeometric test	
**Biological process**	**Raw *P*-value^a^**	**FDR Q-value^b^**	**Fold enrichment^c^**	**FDR Q-value^d^**	**Fold enrichment^e^**

**Genes enriched with adult-only 5hmC blocks**					

Nuclear-transcribed mRNA catabolic process	4.30E-182	3.22E-180	2.49	8.82E-03	1.16

mRNA catabolic process	8.88E-165	5.52E-163	2.25	1.77E-02	1.14

Carbohydrate transport	3.29E-125	1.51E-123	2.06	5.91E-03	1.15

Vesicle localization	9.48E-113	4.05E-111	2.20	3.73E-02	1.16

Fatty acid oxidation	5.52E-102	2.12E-100	2.22	4.59E-03	1.20

Lipid oxidation	1.44E-100	5.39E-99	2.20	3.97E-03	1.20

mRNA 3'-end processing	1.73E-98	6.27E-97	2.24	1.11E-02	1.17

Monocarboxylic acid catabolic process	1.35E-95	4.80E-94	2.21	5.31E-03	1.20

RNA 3'-end processing	2.92E-94	1.02E-92	2.07	2.13E-02	1.15

Fatty acid catabolic process	2.99E-90	9.99E-89	2.22	1.00E-02	1.20

**Genes enriched with fetal-only 5hmC blocks**					

Regulation of granulocyte differentiation	5.88E-23	2.45E-20	6.20	3.56E-02	2.58

Regulation of myeloid cell differentiation	9.74E-17	1.67E-14	2.19	1.84E-03	1.64

Induction of apoptosis by extracellular signals	5.49E-15	7.18E-13	2.18	9.28E-03	1.54

Positive regulation of steroid metabolic process	1.76E-12	1.61E-10	3.87	9.82E-03	2.37

Stem cell development	6.00E-12	5.05E-10	2.09	4.16E-04	2.06

Establishment or maintenance of cell polarity	9.26E-11	6.14E-09	2.02	1.97E-02	1.63

Regulation of protein binding	6.32E-10	3.38E-08	2.03	2.36E-02	1.64

Stem cell maintenance	8.22E-10	4.31E-08	2.00	9.44E-04	2.04

Somatic stem cell maintenance	6.28E-09	2.59E-07	2.15	2.15E-02	2.04

Negative regulation of lipoprotein particle clearance	1.99E-07	6.11E-06	6.52	3.08E-02	3.73

Shown are the top 10 significantly enriched biological processes sorted according to the binomial *P*-values. ^a^Uncorrected *P*-value from the binomial test over genomic regions; ^b^false discovery rate q-value; ^c^fold enrichment of number of genomic regions in the test set; ^d^false discovery rate q-value from the hypergeometric test over genes; ^e^fold enrichment of number of genes in the test set.

### The comparison of human hepatic and cerebellum hydroxymethylomes

We compared our data on fetal and adult hepatic hydroxymethylomes with the previously published 5hmC map of the human adult cerebellum [30]. The study of Szulwach and colleagues [30] revealed 43,272 5hmC-containing genomic intervals in the cerebellum, covering 15.1 Mb. Among them, 8.4 Mb (56%) do intersect with the 5hmC blocks in adult liver. In addition, 3.3 Mb of 5hmC-containing sequences are shared between cerebellum and fetal liver, and 2.7 Mb are common for all these three tissues. Thus, these data indicate that, even though 5hmC distribution is tissue-specific, some 5hmC elements are conserved between different tissues and developmental stages. Pathway analysis of these conserved 5hmC elements reproducibly identified sterol metabolism and insulin response genes among the top 10 pathways (Table S4 in Additional file 1).

## Discussion

Our study reports, for the first time, the characteristics of the 5hmC epigenetic modification in fetal and adult human livers. We have quantified the global 5hmC content in fetal and adult human livers by LC-MS and showed significantly higher levels of 5hmC in adult compared to fetal samples, accompanied by a correspondingly higher expression of *IDH *genes, known 5hmC regulators. Our investigations of the global genomic distribution patterns of 5hmC using NGS categorize this DNA modification as an important mark in hepatic gene expression and development.

Until recently, quantification of 5hmC relied on less precise methods, mainly antibody detection techniques, and utilized a very small sample size. For example, Li and Liu [35] reported the 5hmC human liver content to be 0.45% of total DNA (0.09% of total cytosine), whereas Nestor *et al. *[14] reported values of approximately 0.22% of total cytosines. LC-MS enables sensitive and reliable quantification of 5hmC. Previous studies in murine livers found that 5hmC content quantified by LC-MS was very low (0.05 to 0.07% from the total cytosine) compared to other murine tissues (for example, 0.3 to 0.7% in the central nervous system [26]. Importantly, LC-MS quantification of 5hmC in human liver, to our knowledge, has not been carried out before. Utilizing LC-MS together with a protocol developed to detect both 5mC and 5hmC within the same tissue sample, we were able to quantify the 5mC and 5hmC content in seven adult and eight fetal livers. In contrast to the data reported in mice, our results demonstrate that 5hmC levels in the majority of human adult livers (0.5 to 1% 5hmC) remain comparable to those found in the cerebral cortex (1 to 1.3% 5hmC), the most DNA-hydroxymethylated human tissue identified to date [14,29]. As adult human livers have notably higher global 5hmC content than murine ones, mice are unlikely to serve as relevant models for the human hepatic hydroxymethylome. Moreover, LC-MS quantification of multiple liver samples also demonstrated that the overall content of both 5mC and 5hmC is variable between the samples.

Interestingly, fetal samples showed a significantly lower 5hmC content and very similar levels of total 5mC when compared to adult livers. Drastic differences in 5hmC levels between adult and fetal livers can suggest that DNA hydroxymethylation is an important epigenetic phenomenon for liver development. The observed increase of global 5hmC content in adult compared to fetal livers is interesting because, in the majority of studies, a high 5hmC content has been associated with an embryonic or undifferentiated state of tissues or cells. For example, Ruzov and co-authors [32] report changes of 5hmC abundance in the development of mouse and postulate that 5hmC is enriched in embryonic context compared to adult tissues. Szwagierczak and colleagues [28] showed significantly more 5hmC in undifferentiated mouse embryonic stem cells than in corresponding embryoid bodies, where the decrease in 5hmC content during differentiation was attributed to decreased expression of *TET1*. In addition, reprogramming of differentiated cells into induced pluripotent stem cells activates TET enzymes and leads to accumulation of 5hmC, where the link between 5hmC levels and development was explained by the presence of binding sites for pluripotency-associated transcription factors (OCT4, SOX2) in the promoter of *TET1 *[13,31]. In contrast to these studies, our data indicate a decrease in the extent of 5hmC modifications from the early embryonic stages to adult liver. Similarly, Hahn and colleagues [36] showed that 5hmC is more abundant in terminally differentiated neurons than in neural progenitor cells, thus suggesting that the global 5hmC content increases with terminal maturation in specific tissues.

Our studies revealed that the lower 5hmC content in the fetal livers was accompanied by lower expression of the *IDH1 *and *IDH2 *genes, known regulators of 5hmC level. Hence, it is tempting to speculate that the increase of 5hmC in adult samples is due to the developmental increase of IDH enzymes. However, we did not observe any correlation between the individual 5hmC content and the levels of either *TET1-3 *or *IDH1-2 *transcripts in individual livers (data not shown), indicating that other factors contribute to the global 5hmC content of human liver. Thus, we cannot completely rule out the possibility that the level of TET and/or IDH enzymes can be regulated in a post-transcriptional or post-translational manner, and the developmental burst of 5hmC is controlled by mechanisms other than the increased expression of *IDH1 *and *IDH2 *mRNA.

The observed abundance of 5hmC in adult livers and its preferential localization in actively transcribed genes suggest that bisulfite sequencing data from adult livers should be considered with caution, as bisulfite conversion does not distinguish between 5mC and 5hmC [37]. Hence, specific methods allowing discrimination between these two cytosine modifications become especially important in human liver research.

NGS of 5hmC-enriched genomic DNA obtained from fetal and adult liver samples revealed profound differences in the genomic distribution of 5hmC peaks between the cohorts and significant interindividual variation among samples within the groups. Interestingly, the genomic distribution of 5hmC peaks did not occur at random but appeared to be dependent on the genomic context and on the stage of liver ontogeny. In general, 5hmC is relatively under-represented in repetitive sequences and enriched in coding regions of actively transcribed genes (Figure 5a). In addition, 5hmC peaks are even more enriched in CGI shores (and hence frequently overlap with CGIs), which is in agreement with the tendency of 5hmC to occur in regions with increased CpG density (Figure S4 in Additional file 1). These 5hmC distribution patterns are quite consistent with previous reports for mammalian nervous tissue and embryonic stem cells [13-17,19-21], suggesting a functional role for 5hmC in the regulation of gene expression. In addition, the observed tendency of 5hmC peaks to occur in coding genes and regions with increased CpG density may explain the over-representation of 5hmC blocks on chromosomes 16, 17, 19 and 22 (Figure S2 in Additional file 1). Moreover, the depletion of 5hmC from sex chromosomes as seen here has been observed before in human embryonic stem cells and mouse brain [16,30].

We found that 5hmC blocks in both fetal and adult livers are strongly enriched in genes encoding miRNAs and depleted within lncRNA genes, thus suggesting a role for 5hmC in the regulation of the expression of miRNA genes (Figure 5a). In addition, the observation of high enrichment of 5hmC in hepatic enhancers suggests that 5hmC could play a role in the regulation of gene expression through distal regulatory elements. Moreover, our results evidently indicate the involvement of 5hmC in the developmental expression of protein-coding genes in human liver. Fetal-only 5hmC blocks (that is, genomic intervals that lose 5hmC during liver development, despite the increasing genomic 5hmC content) are especially enriched within genes annotated as belonging to developmental and differentiation-related processes, whereas in adult livers, *de novo *acquired 5hmC appears to predominantly reside in genes associated with hepatic metabolism. These data are in line with the observation of Thomson and co-authors [38], who recently described the dynamics of 5hmC and its effect on gene expression in mouse livers. The authors showed that following exposure to phenobarbital, rapid, dynamic and reciprocal changes in the level of 5hmC and 5mC occurred over the promotor regions of the murine genes known to be transcriptionally regulated by phenobarbital. The changes in hydroxymethylation and methylation further coincided with changes in the histone marks H3K4me2, H3K27me3 and H3K36me3, and indicate that cytosine hydroxymethylation may be crucial in the acute transcriptional regulation of specific hepatic genes.

Here we provide the evidence that adult human liver DNA contains a high level of 5hmC. The brain is another differentiated human tissue that is highly hydroxymethylated [14,29]. We compared our hepatic 5hmC data with the available cerebellum dataset, obtained using the same chemical labeling-based method for 5hmC capture as in our study [30]. The results of pathway analysis of 5hmC intervals that are shared between cerebellum and fetal and adult human livers suggest that the presence of specific 5hmC signatures within genes related to sterol metabolism and insulin response could be conserved between various tissues.

## Conclusions

In this study, we for the first time demonstrate that, in contrast to earlier reports, 5hmC is an abundant epigenetic modification in adult human liver. Our findings on the genomic distribution of 5hmC suggest that this epigenetic mark may be an important determinant of the expression of both protein-coding and miRNA genes in hepatocytes. Furthermore, 5hmC appears to play a significant role in hepatic gene expression changes during liver development. Hence, cytosine hydroxymethylation may be a missing link in the full understanding of the functional as well as the developmental processes of the liver, and also can contribute to interindividual differences in liver function and susceptibility to liver disease. Moreover, our findings suggest the importance of using methods that permit the discrimination between 5mC and 5hmC when investigating the liver epigenome, as the traditional methods based on bisulfite sequencing can cause erroneous conclusions.

## Materials and methods

### Human tissues

Liver samples from fourteen 8- to 12-week-old fetuses were acquired from Karolinska University Hospital (Huddinge, Sweden). Fifty-two liver samples originate from adult organ donors who met accidental death. Of these, 33 were acquired from Karolinska University Hospital, and 19 were commercially purchased from XenoTech (Lenexa, KS, USA) and the International Institute for the Advancement of Medicine (IIAM; Edison, NJ, USA). This work was carried out in compliance with the methods within the Helsinki declaration. The use of fetal and adult liver tissues for the purposes of this study was approved by the Regional Ethics Committee. The reference numbers for the ethical approvals by the Karolinska Institutet Internal Review Ethical Board are 2010/541-23/1, 2010/541-31/1, 2010/678-31/3 and 280/00.

### Nucleic acid purification and quantification

DNA and total RNA were isolated from the liver samples using a AllPrep DNA/RNA/Protein Mini kit (QIAgen, Germantown, MD, USA; catalogue number 80004). DNA was quantified using a Quant-iT PicoGreen dsDNA Assay kit (Invitrogen, Carlsbad, CA, USA; catalogue number P11496) and a SpectraMax Gemini XPS/EM Fluorescence Microplate Reader (Molecular Devices, Sunnyvale, CA, USA). The RNA samples were quantified and assessed for integrity using the Agilent Bioanalyzer 2100 with RNA 6000 Nano kit (Agilent Technologies, Santa Clara, CA, USA; catalogue number 5067-1511). The RIN (RNA integrity number) values of the RNA samples used for cDNA amplification were ≥8.

### Liquid chromatography-mass spectrometry

In order to achieve monomers, 100 ng of each DNA sample were treated with DNA Degradase Plus (ZymoResearch, Irvine, CA, USA; catalogue number E2020) according to the manufacturer's instructions. In parallel, 12 calibration samples containing 0.3125% to 10% 5mC and 0.0625% to 1% 5hmC were prepared from 5-Methylcytosine and 5-Hydroxymethylcytosine DNA Standard Sets (ZymoResearch, catalogue number D5405). The standards were degraded to monomers in the same manner as the DNA samples. Prepared samples were placed into the auto sampler and 5 μl of samples were injected onto the LC-MS system.

Quantification of C, 5mC and 5hmC was accomplished using an Agilent UPLC 1290 system coupled to an Agilent 6460 triple quadrupole (QQQ) equipped with JetStream electrospray ionization (ESI). Chromatographic separation was performed on a Waters Atlantis T3 (C18, 3 μm particles, 100 mm length × 2.1 mm inner diameter; Waters, Milford, MA, USA) at a flow rate of 300 µl/minute and 17°C. Mobile phase A consisted of 10 mM ammonium acetate in MilliQ water, and mobile phase B consisted of 10 mM ammonium formate in methanol with 0.1% (v/v) formic acid. The initial mobile phase composition was 0% of B for 10.2 minutes. From 10.2 to 13.0 minutes, mobile phase B was increased to 20%, and held for 1 minutes. From 14.1 minutes, mobile phase B was decreased to 0% and held until 19.0 minutes.

Mass spectrometric detection was carried out using 6460 QQQ in positive ion multiple reaction monitoring mode. The first quadrupole (Q1) was set to transmit the [M+H]+ ions of the analytes and Q3 was set to transmit selected fragment ions of analytes. Multiple reaction monitoring conditions, such as dwell time, Fragment and CE, are given in Table S1 in Additional file 1. The sheath gas heater was set to 350°C, gas flow was 5 (l/minute) and the sheath gas flow was 10. The instrument was controlled by Mass Hunter Workstation software (Agilent Technologies). 5mC and 5hmC contents were calculated based on the calibration curve constructed by serial standard samples (the standard samples were run twice, before and after real sample analysis). The linear fits of determined area over standard amounts ratio gave R^2 ^values of 0.99981 for 5mC and 0.99941 for 5hmC (Figure S1 in Additional file 1).

### Immunohistochemistry

Sections (40 μm) of snap frozen liver were cut with a microtome cryostat (Cryo-Star HM 560M, MICROM International GmbH, Walldorf, Germany) and fixated in 4% formaldehyde. The denaturation of sections was accomplished by treating the tissues with 1 M HCl for 30 minutes at 37°C. Subsequently, the sections were neutralized by incubating them in 100 mM Tris-HCl (pH 8.5) for 10 minutes. As primary antibodies, a rabbit polyclonal antibody to 5hmC (1:5,000; ActiveMotif, Carlsbad, CA, USA; catalogue number 39769) and a mouse monoclonal antibody to 5mC (1:500; ActiveMotif, catalogue number 39649) were used. For secondary antibodies, we used a goat antibody to rabbit Alexa 647 (1:500; Invitrogen, Carlsbad, CA, USA; catalogue number A21244) and a rabbit antibody to mouse Alexa 488 (1:500; Invitrogen, catalogue number A11059). Samples were counter-stained with the fluorescent nuclear dye 4',6-diamidino-2-phenylindole included in the ProLong^® ^Gold Antifade Reagent mounting media (Invitrogen, catalogue number P-36931). Three-dimensional images were visualized with a Zeiss confocal microscope and ZEN 2009 software. No processing of the images was performed.

### cDNA synthesis and real-time PCR

cDNA was obtained from 1 µg of each total RNA sample using Superscript III First-Strand Synthesis System (Invitrogen, catalogue number 18080-051). Real-time PCR was done with PerfeCTa SYBR Green SuperMix, Low ROX (Quanta Biosciences, Gaithersburg, MD; catalogue number 95056-500) on an ABI 7500 Fast Real-Time PCR System (Applied Biosystems, Foster City, CA, USA). Relative expression values were calculated from Ct values using the ΔΔCt method. The housekeeping gene *EIF2B2 *was used for the normalization of expression values. Primer sequences were published before [34,39]: TET1-F, GCTATACACAGAGCTCACAG; TET1-R, GCCAAAAGAGAATGAAGCTCC; TET2-F, CTTTCCTCCCTGGAGAACAGCTC; TET2-R, TGCTGGGACTGCTGCATGACT; TET3-F, GTTCCTGGAGCATGTACTTC; TET3-R, CTTCCTCTTTGGGATTGTCC; IDH1-F, TCCGTCACTTGGTGTGTAGG; IDH1-R, GGCTTGTGAGTGGATGGGTA; IDH2-F, TGAACTGCCAGATAATACGGG; IDH2-R, CTGACAGCCCCCACCTC; EIF2B2-F, TCAAGATTATCCGGGAGGAG; EIF2B2-R, ATGGAAGCTGAAATCCTCGT.

### Gene expression profiling

The TargetAmp™-Nano Labeling Kit for Illumina^® ^Expression BeadChip (Epicentre Biotechnologies, Madison, WI, USA; catalogue number TAN07924) was used to amplify and biotinylate the RNA samples, according to the manufacturer's instructions. For each sample, 750 ng of biotinylated cRNA were hybridized on Illumina HumanHT-12 v4 BeadChips, according to the standard protocol. The BeadChips were scanned within 24 h using a HiScanSQ scanner. The raw signals were exported using the GenomeStudio software, quantile normalized and further analyzed in R [40].

### Next-generation sequencing

Genomic DNA samples (3 µg in 120 µl) were sonicated using a Covaris S2 with the following settings: duty cycle = 10%, intensity = 5, cycles per burst = 200, time = 6 cycles (60 s each), mode = frequency sweeping, temperature = 4°C. Sonicated DNA was purified with 180 µl of Agencourt AMPure XP beads (Beckman-Coulter, Miami, FL, USA; catalogue number A63880) and assessed for concentration and size distribution with the DNA 1000 kit (Agilent Technologies, catalogue number 5067-1504) on an Agilent Bioanalyzer 2100. All samples manifested a size distribution between 100 and 250 bp, with peak at 150 to 200 bp.

Sheared DNA samples were enriched for the 5hmC-containing fraction with the Hydroxymethyl Collector kit (ActiveMotif, catalogue number 55013) following the manufacturer's instructions. In parallel, non-enriched aliquots of sheared DNA were diluted 1:100. 5hmC-enriched samples and their non-enriched counterparts were then ligated to TruSeq adapters and PCR amplified according to the protocol detailed in Text S1 in Additional file 1. The resultant NGS libraries were assessed for size distribution with the DNA High Sensitivity kit (Agilent Technologies, catalogue number 5067-4626) on an Agilent Bioanalyzer 2100 and quantified using a Agilent QPCR NGS Library Quantification kit for Illumina Genome Analyzer (Agilent Technologies, catalogue number G4880A). The 5hmC-enriched adult and fetal samples were pooled separately and sequenced on two separate lanes. The control non-enriched samples were also pooled separately for the fetal and adult samples, and each pool was sequenced on seven lanes. The flowcells (v3) were sequenced on an Illumina HiSeq2000 using paired-end sequencing of 100 bp and 6 bp for the index read.

### Sequencing data analysis

The FASTQ sequences of all samples were aligned to the reference human genome (NCBI37/hg19) using Burrows-Wheeler Aligner (BWA) [41]. Parameter -q 20 was used for read trimming, and all reads with mapping quality <20 were filtered out. Duplicate reads generated during the PCR amplification were removed using SAMtool [42]. 5hmC peak identification was performed using MACS with the following parameters: tag size = 100; effective genome size = 2.70e+09; band width = 200; model fold = 10, 30; *P*-value cutoff = 1.00e-05; ranges for calculating regional lambda = 1,000 bp and 10,000 bp [43]. The data discussed in this publication have been deposited in NCBI's Gene Expression Omnibus and are accessible through GEO Series accession number GSE49291 [44].

All subsequent steps of data analysis were done using custom Python3 scripts, which are available on request. The coordinates of genomic features were downloaded from the UCSC Table Browser (tables refGene, lincRNAsTranscripts, cpgIslandExt, rmsk, wgEncodeBroadHmmHepg2HMM). The coordinates of 5hmC peaks in human cerebellum were downloaded as Supplementary Data Set 10 of the original paper of Szulwach and co-authors [30] and converted to Hg19 using the Galaxy LiftOver tool [45]. The relative enrichment of 5hmC elements in different functional categories of genes was evaluated using the Genomic Regions Enrichment of Annotations Tool (GREAT) [46], setting the basal gene regulatory domains within 5 Kb upstream and 1 Kb downstream of the transcription start site, and up to 1 Mb extended regulatory domains in both directions to the nearest gene's basal domain [47].

### Locus-specific validation of next-generation sequencing data

To validate NGS data at single-base resolution in selected genomic intervals, we employed the TAB-Seq method [48]. Briefly, 1 µg of DNA was oxidized with the WiseGene 5hmC TAB-Seq kit (WiseGene, Chicago, IL, USA) following the manufacturer's instructions [49]. Then, both the oxidized DNA and the corresponding untreated 500 ng aliquots were bisulfite converted with EZ DNA Methylation-Gold kit (ZymoResearch, catalogue number D5005), and the resultant DNA samples were amplified with the following primers: DROSHA-F, TTTAGTTGGGTGGTTTTATTTG; DROSHA-R, CAACTACTTTTATACCAAC; CDH2-F, GTGGTAGTGGTTGTAATTATATA; CDH2-R, CTTAAAAAATAAATCATTCCTCCC. To increase the specificity of amplification, the first-round PCR products were purified, diluted 1:10,000 and amplified again with the nested primers: DROSHA(n)-F, GTTTTATGTTTTGTGGTAGA; DROSHA(n)-R, ACTTTTATACCAACCTAACA; CDH2(n)-F, ATGAGAAGAGTTATGATATGGGAAT; CDH2(n)-R, AAAAATAAATCATTCCTCCC. The second-round PCR products were purified and subjected to direct Sanger sequencing (Eurofins MWG Operon, Ebersberg, Germany) [50]. The C/(C+T) ratios from the oxidized samples reflect the 5hmC values, whereas such ratios from corresponding non-oxidized aliquots are equal to the sum of 5mC and 5hmC at the same CpG sites. Hence, the subtraction of the resultant C/(C + T ratios) permits the calculation the 5mC and 5hmC values for each CpG site.

## Abbreviations

5fC: 5-formylcytosine; 5mC: 5-methylcytosine; 5hmC: 5-hydroxymethylcytosine; bp: base pair; CGI: CpG island; IDH: isocitrate dehydrogenase; LC-MS: liquid chromatography-mass spectrometry; lncRNA: long non-coding RNA; miRNA: microRNA; NGS: next-generation sequencing.

## Competing interests

The authors declare that they have no competing interests.

## Authors' contributions

MI conceived the study, carried out sample preparation and data interpretation and drafted the manuscript. MKal carried out the data analysis. MKac participated in the design of the study and in sample preparation. IB carried out the immunohistochemistry assays and the locus-specific validation of NGS data. KK carried out the LC-MS analysis. AR provided the human fetal liver samples. LM carried out the next-generation sequencing and microarray assays. AM, LM and MIS participated in planning of the study and coordinated and financed the work from their grants. All authors carried out manuscript revisions. All authors read and approved the final manuscript.

## Supplementary Material

Additional file 1 **Text S1 (the detailed NGS library preparation protocol), Figures S1 to S5 and Tables S1 to S4**. Click here for file

Additional file 2 **Tab-delimited text file describing all 5hmC blocks discovered in the epigenomes of fetal livers**. Fields 1 to 3 contain genomic coordinates (Hg19) of each 5hmC block. Field 4 indicates the number of 5hmC-positive samples in a given age cohort (that is, those samples that manifest a 5hmC peak in a given genomic interval). Fields 5 and 6 provide some metrics of 5hmC occupancy of given 5hmC blocks; field 5 indicates the sum of 5hmC peak lengths (bp), whereas field 6 shows the sum of NGS reads in 5hmC peaks among all 5hmC-positive samples. Click here for file

Additional file 3 **Tab-delimited text file describing all 5hmC blocks discovered in the epigenomes of adult human livers**. Fields 1 to 3 contain genomic coordinates (Hg19) of each 5hmC block. Field 4 indicates the number of 5hmC-positive samples in a given age cohort (that is, those samples that manifest a 5hmC peak in a given genomic interval). Fields 5 and 6 provide some metrics of 5hmC occupancy of given 5hmC blocks; field 5 indicates the sum of 5hmC peak lengths (bp), whereas field 6 shows the sum of NGS reads in 5hmC peaks among all 5hmC-positive samples. Click here for file
