# Supplementary material for: Ontogeny, distribution and potential roles of 5-hydroxymethylcytosine in human liver function
Source: Genome Biol. 2013 Aug 19;14(8):R83. doi: 10.1186/gb-2013-14-8-r83 (PMC4054829; doi:10.1186/gb-2013-14-8-r83)
Supplement: Additional file 1 — Text S1 (the detailed NGS library preparation protocol), Figures S1 to S5 and Tables S1 to S4. [file gb-2013-14-8-r83-S1.DOC]

**Text S1.** **The detailed NGS libraries preparation protocol**

Input DNA: 10 ng of sonicated gDNA (either 5hmC-enriched or non-enriched);

Materials requires:

- TruSeq adapters (0.3 pmol/µl):

diluted 1:50 with 0.1x TE from TruSeq DNA Sample Prep kit v2 (Illumina Inc., Cat. # FC-121-2001);

- TruSeq PCR Primers 1.0 and 2.0 (25 pmol/µl; from TruSeq DNA Sample Prep kit v2);

- NEBNext End Repair Module (New England Biolabs, Cat. # E6050S);

- NEBNext dA-Tailing Module (New England Biolabs, Cat. # E6053S);

- NEBNext Quick Ligation Module (New England Biolabs, Cat. # E6056S);

- Agencourt AMPure XP beads (Beckman-Coulter, Cat. # A63880);

- MinElute Reaction Cleanup kit (QIAgen, Cat. # 28204);

- Herculase II Fusion DNA polymerase (Agilent, Cat. # 600675);

- QPCR NGS Library Quantification Kit for Illumina Genome Analyzer (Agilent Technologies, Cat. #G4880A)

- Nuclease-Free Water (QIAgen, Cat. #129114).

1) Dilute 10 ng DNA with water to 43 µl, mix with 5 µl NEBNext End Repair reaction buffer and 2 µl NEBNext End Repair Enzyme mix. Incubate at room temperature for 30 min;

2) Purify with MinElute Reaction Cleanup kit (elute in 21 µl water);

3) Mix 21 µl eluate with 2.5 µl NEBNext dA-Tailing Reaction Buffer and 1.5 µl NEBNext Klenow Fragment. Incubate at 37°C for 30 min;

4) Purify with MinElute Reaction Cleanup kit (elute in 16 µl water);

5) Mix 16 µl eluate with 5 µl NEBNext Quick Ligation buffer, 1.5 µl TruSeq adapter (0.3 pmol/µl) and 2.5 µl NEBNext Quick Ligase. Incubate at room temperature for 15 min;

6) Add 5 µl 0.5 M EDTA, pH 8.0;

7) Purify with 30 µl of Agencourt AMPure XP beads, elute in 50 µl water;

8) Purify one more time with 50 µl of Agencourt AMPure XP beads, elute in 50 µl water;

9) Split adapter-ligated DNA into 2 aliquots;

10) Dilute 1^st^ aliquot to 37 µl, mix with 1 µl TruSeq PCR Primer 1.0, 1 µl TruSeq PCR Primer 2.0, 10 µl Herculase II Buffer, 0.5 µl dNTPs mix (25 mM each) and 1 µl Herculase II polymerase. Amplify with the following program:

95°C, 5' (1 cycle)

98°C, 30” (1 cycle)

98°C, 10”; 63°C, 30”; 72°C, 30” (14 cycles)

72°C, 5’;

11) Purify with 50 µl of Agencourt AMPure XP beads, elute in 30 µl water;

12) Assess concentration of NGS libraries by Agilent Bioanalyzer 2100 (DNA High Sensitivity kit) and/or Agilent QPCR NGS Library Quantification Kit. If concentration of libraries is too high or too low, then repeat PCR amplification with the remaining aliquot of adapter-ligated DNA, using either less or more cycles.

**Figure S1A. Calibration curves used for 5mC and 5hmC quantitation**.

1) Calibration curve of 5mC:


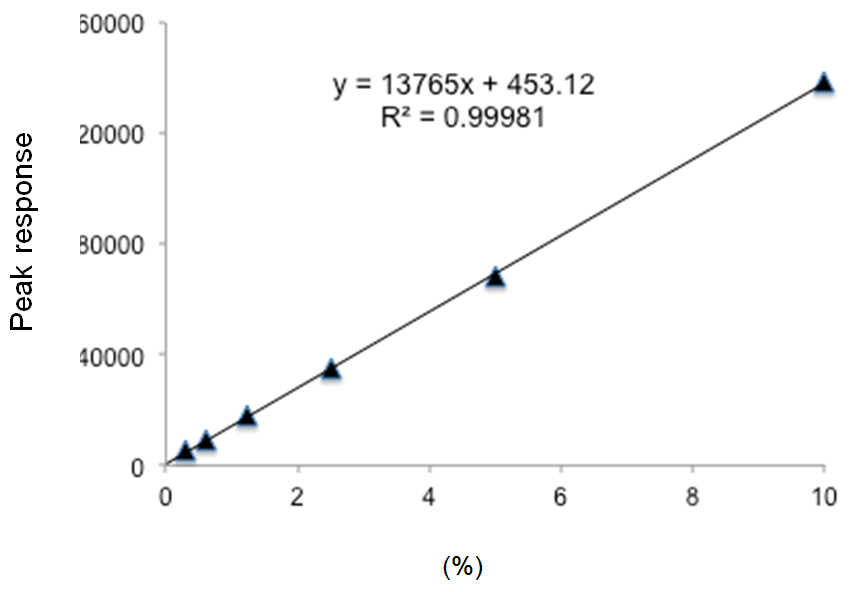


2) Calibration curve of 5hmC:


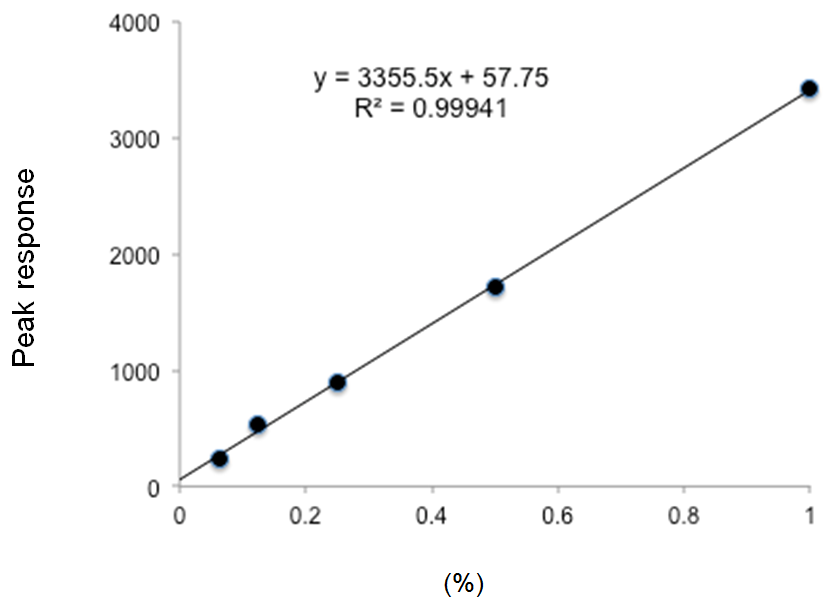


**Figure S1B. Reproducibility of calibration curves.**

1) Reproducibility of calibration curves of 5mC:


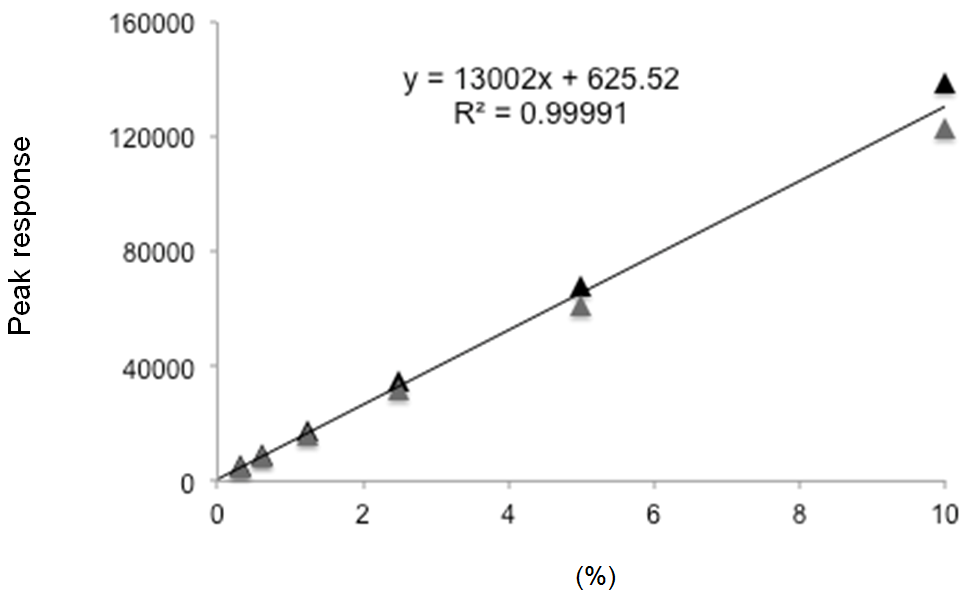


2) Reproducibility of calibration curves of 5hmC:


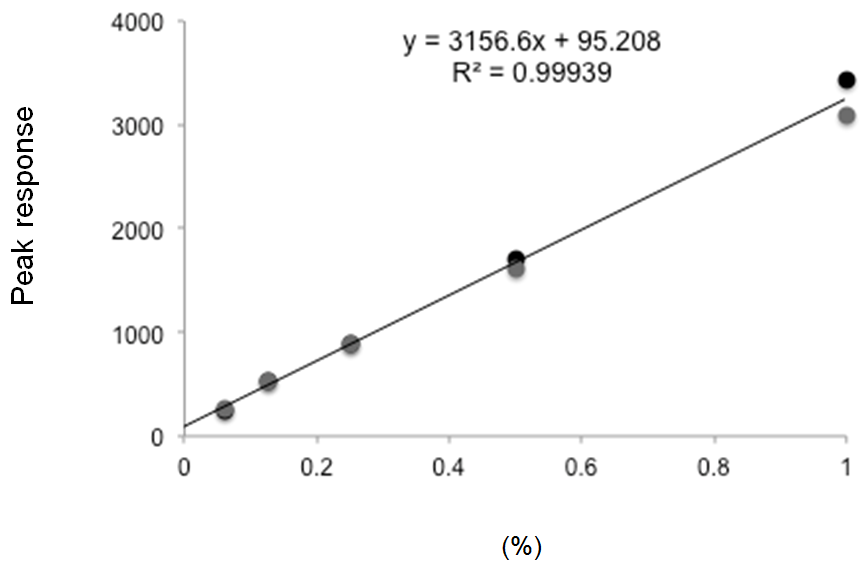


(n = 2, linear regression based on average peak response)

**Figure S2. The chromosomal distribution of 5hmC, CpG and gene densities.**


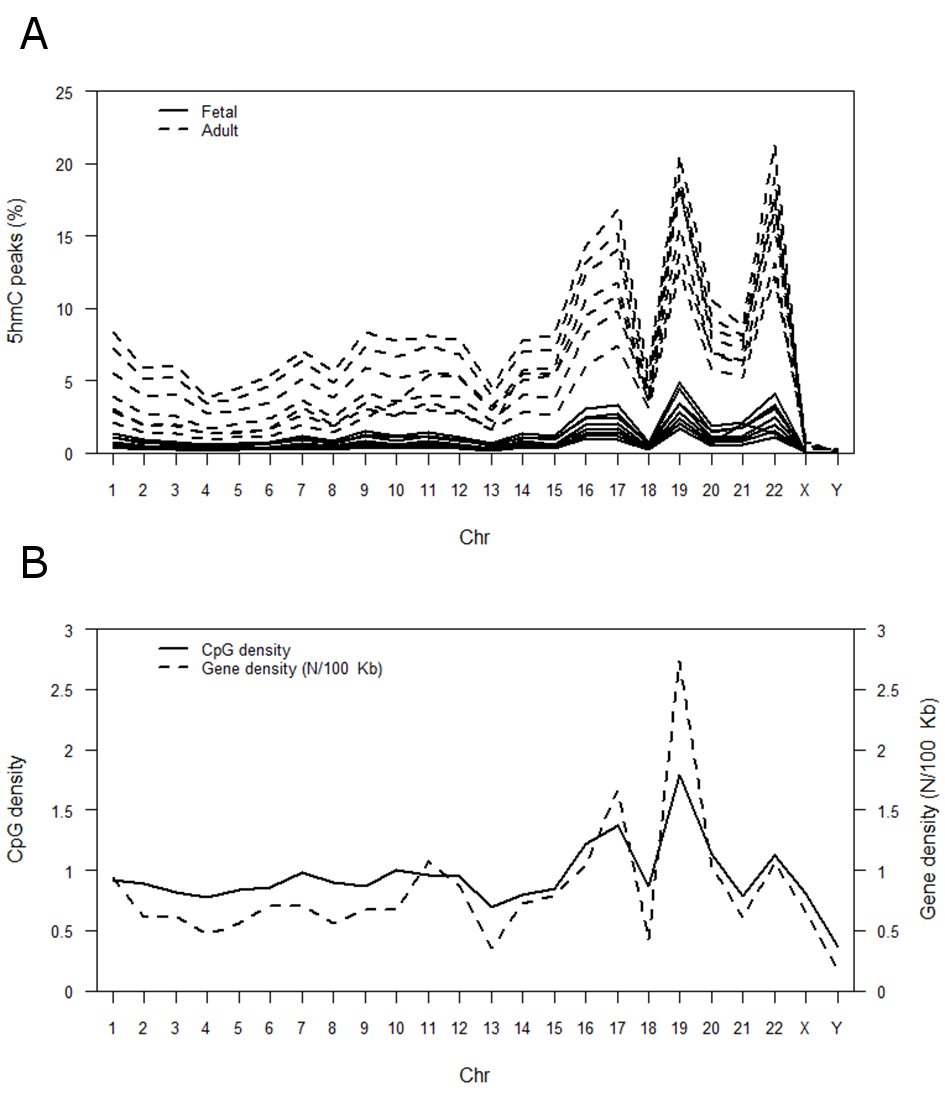


(A) The distibution of 5hmC peaks among chromosomes. The Y-axis shows the percentage of chromosome length which is occupied by 5hmC peaks in each fetal or adult sample. (B) The distribution of CpG density (expressed as the mean number of CpG sites per 100 bp nucleotide sequence) and gene density (expressed as the mean number of genes per 100 Kb of nucleotide sequence) among chromosomes.

**Figure S3. The fractions of peaks which are shared between samples.**


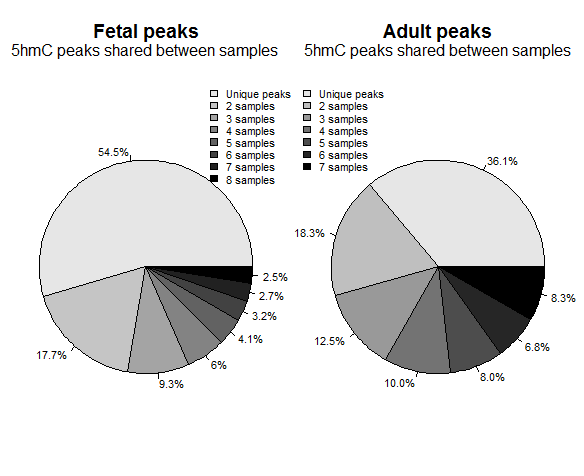


**Figure S4. Boxplots of CpG density of fetal and adult 5hmC blocks and selected genomic features.**

**
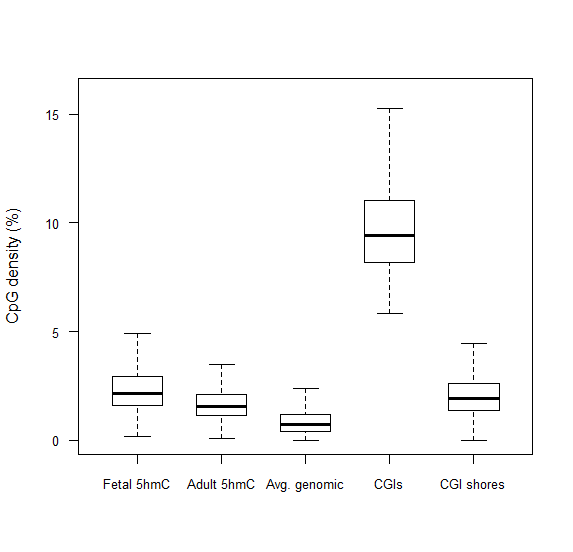
**

CpG density was expressed as the number of CpG sites per 100 bp of nucleotide sequence. The bars indicate 5% and 95% quantiles.

**Figure S5A. The validation of NGS data at 4 CpG sites in the *DROSHA* gene**

**
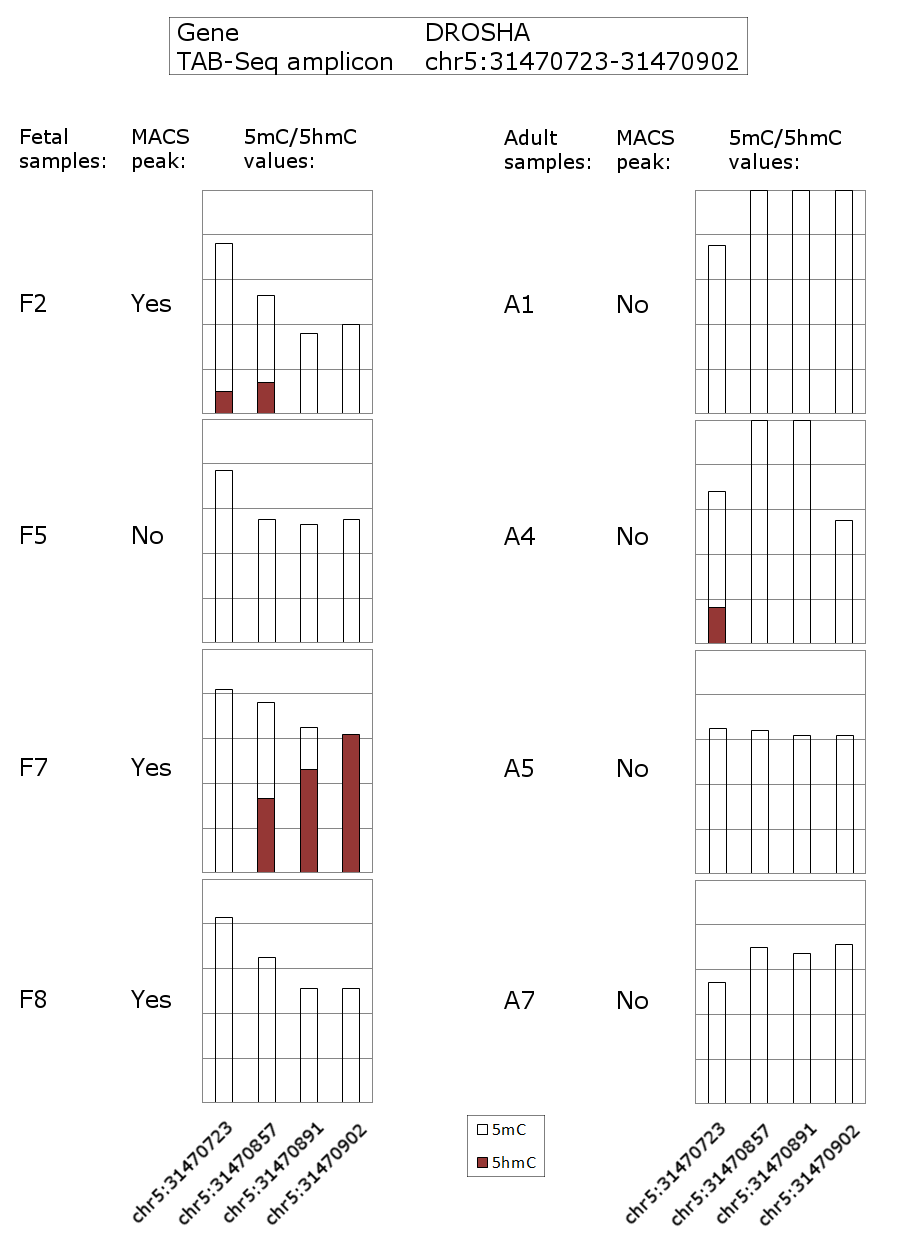
**

**Figure S5B. The validation of NGS data at 7 CpG sites in the *CDH2* gene**

**
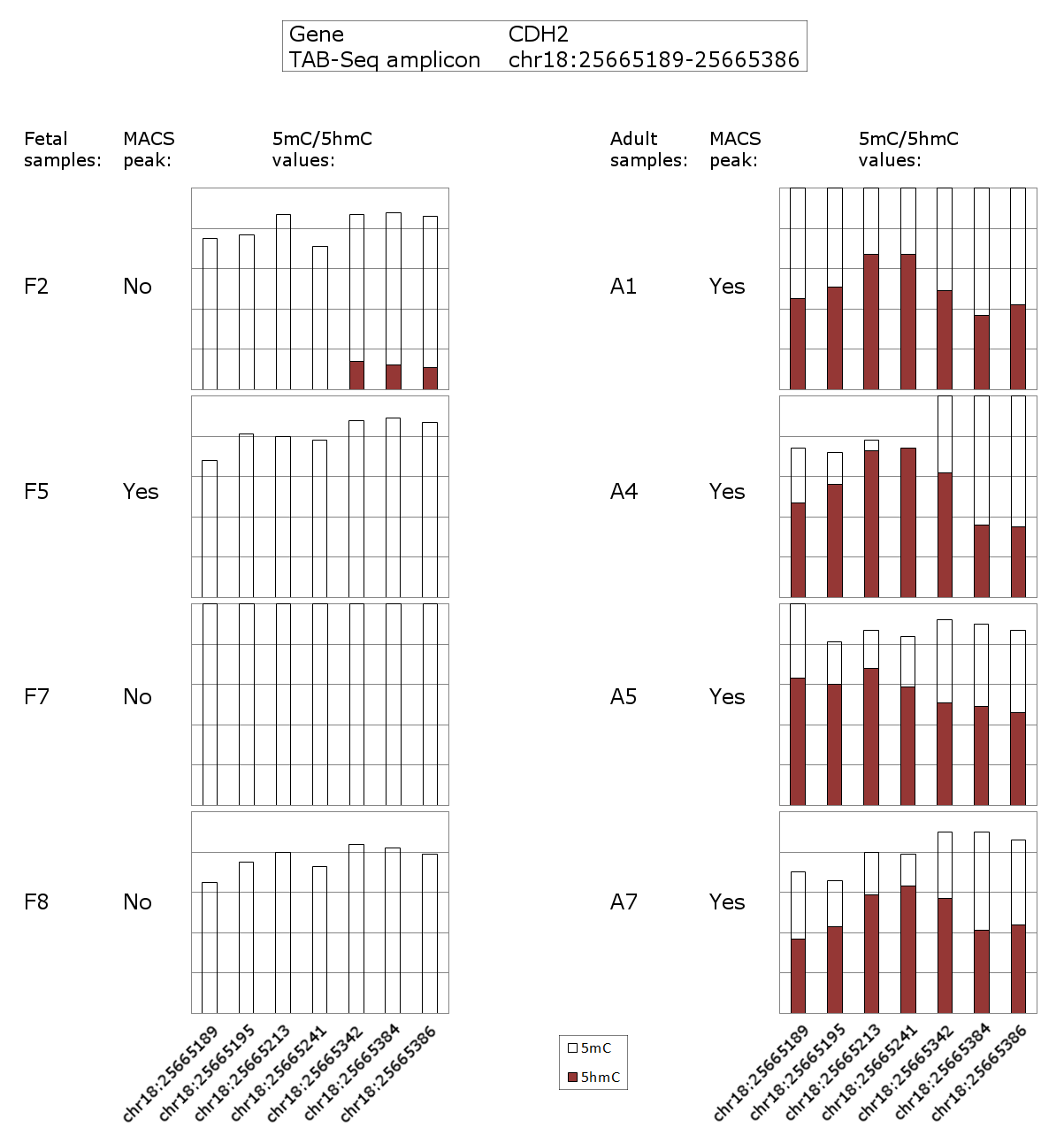
**

**Table S1. Conditions for LC-MS analysis of 5mC and 5hmC**

| Analyte | RT (min) | Reaction | Dwell (msec) | Fragment (V) | CE (V) |
| --- | --- | --- | --- | --- | --- |
| dC | 8.42 | 228.1 🡪 112.1 | 200 | 120 | 12 |
| 5mC | 13.61 | 242.1 🡪 126.1 | 200 | 126 | 34 |
| 5hmC | 10.83 | 258.1 🡪 142.1 | 200 | 135 | 20 |

**Table S2**

LC-MS quantification of global 5mC and 5hmC content in 12 control samples and 15 human liver gDNA samples

|  | Measured content of: | |
| --- | --- | --- |
| Sample | 5mC | 5hmC |
| Fetal sample 1 | 2.7694% | 0.0115% |
| Fetal sample 2 | 4.2116% | 0.0395% |
| Fetal sample 3 | 7.6822% | 0.0543% |
| Fetal sample 4 | 3.2915% | 0.0206% |
| Fetal sample 5 | 4.2549% | 0.1186% |
| Fetal sample 6 | 5.4666% | 0.0524% |
| Fetal sample 7 | 4.1094% | 0.0704% |
| Fetal sample 8 | 4.7623% | 0.0396% |
| Adult sample 1 | 4.8535% | 0.4913% |
| Adult sample 2 | 6.2943% | 1.0275% |
| Adult sample 3 | 3.5223% | 0.2025% |
| Adult sample 4 | 4.2790% | 0.6213% |
| Adult sample 5 | 7.1488% | 0.7347% |
| Adult sample 6 | 5.2680% | 0.8164% |
| Adult sample 7 | 5.7444% | 0.3140% |

5hmC values in red are below the limit of quantification (0.0625%)

**Table S3.**

Quality metrics of next-generation sequencing and main statistics on 5hmC peaks (**fetal samples**)

| Liver sample | DNA sample | Reads sequenced | Reads mapped | Mapping efficiency | Mapped reads with MAPQ>=20 | Duplicate reads | Valid reads (after duplicate removal) | Number of called peaks | Median peak length, bp | Sum of peak length, Mb |
| --- | --- | --- | --- | --- | --- | --- | --- | --- | --- | --- |
| Fetal sample 1 | 5hmC enriched | 108 604 072 | 104 054 380 | 96% | 98 741 508 | 50% | 49 758 622 | 11 802 | 990 | 13.33 |
|  | Genomic control | 270 408 000 | 259 306 634 | 96% | 244 446 803 | 83% | 41 128 882 |  |  |  |
| Fetal sample 2 | 5hmC enriched | 107 834 486 | 103 419 189 | 96% | 98 290 235 | 50% | 49 171 974 | 17 237 | 872 | 16.82 |
|  | Genomic control | 327 484 120 | 313 816 050 | 96% | 296 654 787 | 75% | 74 014 205 |  |  |  |
| Fetal sample 3 | 5hmC enriched | 65 824 072 | 62 796 505 | 95% | 59 486 169 | 32% | 40 613 552 | 11 366 | 766 | 9.51 |
|  | Genomic control | 204 317 128 | 195 681 026 | 96% | 184 315 812 | 53% | 86 445 353 |  |  |  |
| Fetal sample 4 | 5hmC enriched | 59 554 788 | 56 652 941 | 95% | 53 635 452 | 18% | 43 976 729 | 12 406 | 881 | 12.21 |
|  | Genomic control | 298 605 366 | 285 463 004 | 96% | 268 460 403 | 62% | 102 650 157 |  |  |  |
| Fetal sample 5 | 5hmC enriched | 79 636 492 | 76 117 742 | 96% | 72 909 357 | 26% | 53 707 576 | 27 132 | 887 | 27.23 |
|  | Genomic control | 266 449 648 | 254 060 575 | 95% | 240 454 916 | 43% | 137 249 067 |  |  |  |
| Fetal sample 6 | 5hmC enriched | 98 878 810 | 93 684 076 | 95% | 88 584 240 | 20% | 71 142 596 | 16 734 | 1 009 | 19.55 |
|  | Genomic control | 279 926 598 | 268 420 421 | 96% | 252 683 858 | 63% | 94 509 761 |  |  |  |
| Fetal sample 7 | 5hmC enriched | 73 230 026 | 70 161 598 | 96% | 66 869 194 | 9% | 61 012 436 | 32 522 | 908 | 33.85 |
|  | Genomic control | 194 381 148 | 186 372 572 | 96% | 175 999 752 | 30% | 123 676 073 |  |  |  |
| Fetal sample 8 | 5hmC enriched | 74 279 410 | 70 648 778 | 95% | 67 049 027 | 11% | 60 007 438 | 26 193 | 915 | 27.38 |
|  | Genomic control | 190 518 788 | 182 124 937 | 96% | 171 796 758 | 20% | 137 797 575 |  |  |  |

**Table S3 (continued)**

Quality metrics of next-generation sequencing and main statistics on 5hmC peaks (**adult samples**)

| Liver sample | DNA sample | Reads sequenced | Reads mapped | Mapping efficiency | Mapped reads with MAPQ>=20 | Duplicate reads | Valid reads (after duplicate removal) | Number of called peaks | Median peak length, bp | Sum of peak length, Mb |
| --- | --- | --- | --- | --- | --- | --- | --- | --- | --- | --- |
| Adult sample 1 | 5hmC enriched | 81 556 096 | 78 181 011 | 96% | 74 279 405 | 13% | 64 762 089 | 88 989 | 1 281 | 141.37 |
|  | Genomic control | 282 534 238 | 266 101 485 | 94% | 248 618 110 | 80% | 49 649 210 |  |  |  |
| Adult sample 2 | 5hmC enriched | 93 155 208 | 88 219 818 | 95% | 83 210 011 | 7% | 77 250 884 | 68 779 | 1 271 | 106.46 |
|  | Genomic control | 227 753 038 | 214 028 019 | 94% | 147 215 062 | 55% | 65 669 676 |  |  |  |
| Adult sample 3 | 5hmC enriched | 72 832 628 | 69 005 305 | 95% | 65 140 780 | 4% | 62 733 384 | 72 255 | 1 008 | 92.4 |
|  | Genomic control | 241 616 238 | 227 095 136 | 94% | 126 797 715 | 42% | 73 499 935 |  |  |  |
| Adult sample 4 | 5hmC enriched | 82 356 338 | 78 816 786 | 96% | 74 779 676 | 6% | 70 522 110 | 134 956 | 1 045 | 179.42 |
|  | Genomic control | 261 145 256 | 245 879 138 | 94% | 229 515 496 | 53% | 108 515 090 |  |  |  |
| Adult sample 5 | 5hmC enriched | 107 883 220 | 102 974 353 | 95% | 97 652 796 | 8% | 90 198 355 | 76 434 | 1 186 | 110.65 |
|  | Genomic control | 249 230 115 | 244 487 445 | 98% | 230 512 262 | 48% | 120 405 609 |  |  |  |
| Adult sample 6 | 5hmC enriched | 52 535 882 | 50 303 377 | 96% | 47 743 318 | 10% | 43 071 763 | 72 326 | 949 | 80.25 |
|  | Genomic control | 249 608 846 | 244 735 761 | 98% | 230 059 982 | 49% | 116 387 484 |  |  |  |
| Adult sample 7 | 5hmC enriched | 142 525 212 | 136 470 704 | 96% | 129 558 847 | 27% | 94 527 438 | 131 448 | 1 157 | 203.05 |
|  | Genomic control | 263 980 036 | 248 783 848 | 94% | 232 004 702 | 49% | 118 363 037 |  |  |  |

**Table S4. Functional analysis of common 5hmC-containing intervals between cerebellum, fetal and adult livers.**

A. 5hmC intervals conserved between cerebellum and adult livers (n = 22,706; 8.4 Mb):

| Biological process | Binom Raw P-Value | Binom FDR Q-Val | Binom Fold Enrichment | Hyper FDR Q-Val | Hyper Fold Enrichment |
| --- | --- | --- | --- | --- | --- |
| **sterol metabolic process** | 7.26e-101 | 1.25e-98 | 2.90 | 3.45e-03 | 1.48 |
| negative regulation of sequence-specific DNA binding transcription factor activity | 1.22e-86 | 1.43e-84 | 2.50 | 8.16e-03 | 1.48 |
| **cholesterol metabolic process** | 9.76e-83 | 1.06e-80 | 2.73 | 1.24e-03 | 1.53 |
| **regulation of insulin receptor signaling pathway** | 7.53e-80 | 7.58e-78 | 4.68 | 2.16e-02 | 1.90 |
| regulation of generation of precursor metabolites and energy | 1.73e-71 | 1.30e-69 | 3.10 | 4.91e-02 | 1.54 |
| regulation of ARF protein signal transduction | 1.06e-58 | 5.37e-57 | 2.93 | 3.38e-02 | 1.59 |
| negative regulation of cellular catabolic process | 1.23e-43 | 3.61e-42 | 2.83 | 2.06e-02 | 1.74 |
| endothelial cell differentiation | 1.16e-35 | 2.54e-34 | 2.41 | 1.94e-02 | 1.77 |
| histone methylation | 3.95e-34 | 8.34e-33 | 2.18 | 4.66e-02 | 1.52 |
| protein methylation | 4.66e-34 | 9.77e-33 | 2.03 | 1.88e-02 | 1.48 |

B. 5hmC intervals conserved between cerebellum and fetal livers (n = 8,449; 3.3 Mb):

| Biological process | Binom Raw P-Value | Binom FDR Q-Val | Binom Fold Enrichment | Hyper FDR Q-Val | Hyper Fold Enrichment |
| --- | --- | --- | --- | --- | --- |
| regulation of lipid metabolic process | 3.13e-66 | 7.02e-64 | 2.61 | 4.82e-03 | 1.58 |
| cellular response to peptide hormone stimulus | 9.53e-58 | 1.33e-55 | 2.28 | 3.35e-04 | 1.58 |
| **sterol metabolic process** | 1.46e-56 | 1.91e-54 | 3.40 | 5.65e-03 | 1.75 |
| **cholesterol metabolic process** | 2.60e-56 | 3.35e-54 | 3.45 | 5.58e-03 | 1.78 |
| **cellular response to insulin stimulus** | 5.96e-53 | 6.37e-51 | 2.41 | 5.65e-03 | 1.54 |
| regulation of lipid biosynthetic process | 4.97e-45 | 3.69e-43 | 2.96 | 4.58e-02 | 1.69 |
| protein kinase B signaling cascade | 2.70e-43 | 1.83e-41 | 7.96 | 3.79e-02 | 2.79 |
| regulation of skeletal muscle fiber development | 1.25e-42 | 8.31e-41 | 3.18 | 1.18e-02 | 2.33 |
| **response to insulin stimulus** | 6.76e-41 | 4.05e-39 | 2.02 | 2.79e-03 | 1.51 |
| lens fiber cell differentiation | 4.16e-40 | 2.37e-38 | 4.36 | 3.10e-02 | 2.56 |
| **regulation of insulin receptor signaling pathway** | 4.91e-39 | 2.62e-37 | 5.30 | 5.00e-04 | 3.04 |
| negative regulation of glial cell proliferation | 9.23e-39 | 4.78e-37 | 6.83 | 4.83e-02 | 3.49 |

C. 5hmC intervals conserved between cerebellum, fetal and adult livers (n = 7,589; 2.6 Mb):

| Biological process | Binom Raw P-Value | Binom FDR Q-Val | Binom Fold Enrichment | Hyper FDR Q-Val | Hyper Fold Enrichment |
| --- | --- | --- | --- | --- | --- |
| blood coagulation | 6.67e-71 | 2.16e-68 | 2.02 | 4.40e-03 | 1.36 |
| hemostasis | 7.41e-71 | 2.32e-68 | 2.02 | 3.07e-03 | 1.36 |
| regulation of lipid metabolic process | 2.62e-69 | 6.19e-67 | 2.76 | 1.63e-02 | 1.56 |
| cellular response to peptide hormone stimulus | 9.39e-60 | 1.58e-57 | 2.38 | 2.33e-03 | 1.55 |
| **cellular response to insulin stimulus** | 1.34e-55 | 1.72e-53 | 2.55 | 9.12e-03 | 1.56 |
| response to UV-A | 5.60e-54 | 6.37e-52 | 16.04 | 4.44e-02 | 5.13 |
| **sterol metabolic process** | 8.16e-53 | 9.05e-51 | 3.45 | 1.76e-02 | 1.73 |
| **cholesterol metabolic process** | 2.84e-52 | 2.99e-50 | 3.49 | 2.00e-02 | 1.75 |
| white fat cell differentiation | 6.29e-52 | 6.34e-50 | 7.88 | 4.96e-02 | 3.27 |
| protein kinase B signaling cascade | 1.00e-43 | 7.59e-42 | 8.52 | 2.35e-02 | 3.08 |
